# Cytological Study of Topical Effect of Azelastine Hydrochloride on the Nasal Mucous Membrane Cells in Various Nasal Rhinitis Types

**DOI:** 10.3390/cells12232697

**Published:** 2023-11-24

**Authors:** Ewa Trybus, Wojciech Trybus, Teodora Król

**Affiliations:** Department of Medical Biology, Jan Kochanowski University of Kielce, Uniwersytecka 7, 25-406 Kielce, Poland; teodora.krol@ujk.edu.pl

**Keywords:** nasal cytology, rhinitis, azelastine hydrochloride

## Abstract

Previous reports on the benefits of using local therapy with azelastine in rhinitis focus on the assessment of clinical symptoms and the analysis of nasal lavage for the presence of inflammatory cells and the expression of adhesion molecules. Little attention has been paid to studies assessing the effect of azelastine on individual cytotypes of the nasal mucosa, especially epithelial cells, also in the context of inducing morphological changes. The aim of this study was the cytological analysis of swabs taken from the surface of the nasal mucosa of patients with allergic rhinitis (AR) and nonallergic/vasomotor rhinitis (NAR/VMR) who were subjected to 4 weeks of therapy with azelastine and then comparing the obtained results with the pre-treatment condition. The technique of obtaining materials for cytoanalysis included sampling, staining of smears, microscopic analysis, and preparation of cytograms. Our studies confirmed the therapeutic benefits of azelastine in both study groups. Significant changes were demonstrated, confirming the regeneration of ciliated cells and the induction of autophagy and apoptosis in epithelial cells. Such changes indicate new mechanisms of action of azelastine, which play a significant role in restoring homeostasis in the nasal mucosa. The presented research also results in a detailed description of cytological changes in both studied rhinitis types, which complements the knowledge regarding prognostic indicators.

## 1. Introduction

Azelastine hydrochloride, a representative of the second generation of H1 receptor antagonists, is used in the basic therapy of rhinitis, such as perennial and seasonal allergic rhinitis (also in the course of asthma and COPD) and perennial nonallergic rhinitis, as well as in the prevention and treatment of allergic conjunctivitis [1,2,3,4]. Azelastine is believed to affect the course of both the early and late phases of the allergic reaction, which is due to the multidirectional nature of its action involving not only antihistamine mechanisms but also anti-allergic and anti-inflammatory ones [5]. The non-receptor effect of azelastine [6,7,8,9] provides a broad perspective for discovering new mechanisms of action of this compound, and thus new therapeutic possibilities. Recent in vitro studies have demonstrated the ability of azelastine to prevent and inhibit SARS-CoV-2 infection in nasal tissue [10].

Azelastine shows high clinical efficacy and very good tolerability in adults and children in both systemic and topical administration [11,12,13,14]. However, the intranasal form of administration allows obtaining a high concentration of the compound directly at the site of the ongoing inflammatory process and serves to limit possible side effects caused by systemic exposure [5]. Moreover, topical azelastine shows better therapeutic effects in relieving the symptoms of allergic rhinitis compared to oral antihistamines [3,15,16] and also shows a faster onset of action than intranasal corticosteroid preparations [17,18].

Previous reports on the cytological evaluation of the effects of topical azelastine hydrochloride therapy in rhinitis are based on data obtained from the analysis of lavage collected from patients after allergen provocation, carried out for the effect of this compound on the number of inflammatory cells and the expression of adhesion molecules [16]. The lavage method allows a high number of cells loosely associated with the nasal mucosa to be obtained—inflammatory cells, with the exception of basophils and mast cells, the absence of which is explained by the complicated method of obtaining samples, which promotes cell damage. In contrast, the percentage of epithelial cells identified in nasal lavage is very low [19]. The highest percentage of epithelial cells is obtained by the swab technique (exfoliative cytology-NC), which provides more information about living epithelial cells compared to other cytological methods. In addition, the area of swab collection is relatively larger compared to biopsy [20].

The present study aimed to analyze the effect of local azelastine hydrochloride therapy on nasal mucosal cells of patients with chronic symptoms of allergic rhinitis (AR) and nonallergic rhinitis (NAR) using material obtained by exfoliative cytology. Both before and after azelastine treatment, the cellular composition of the nasal epithelium as well as the morphological changes in individual cells were analyzed in detail. Columnar cells, which constitute the first line of defense and are the first to respond to various factors, were thoroughly evaluated.

## 2. Materials and Methods

### 2.1. Study Design and Participants

The research was performed in cooperation with an allergist from the Allergy Clinic, Military Specialist Medical Clinic SP ZOZ in Kielce. The study was accepted by the Jan Kochanowski University in Kielce Bioethical Committee (No. 45/2011). The research period covered the months of April–June. Nasal swabs (after obtaining informed consent) were performed during a routine visit to a doctor’s office in 40 patients (14 girls and 26 boys) aged 7–14 years (median 11.5 years) with symptoms of chronic rhinitis. The study was conducted during severe clinical symptoms of rhinitis, at least 1 week after drug discontinuation. Nasal cytology was also performed in 10 healthy people (5 women and 5 men, aged 5–22 years, median 14.20 years), without symptoms of rhinitis, who constituted the control group. The results of the control group corresponded to the description of healthy nasal mucosa [21]. Based on the cytograms and comparison of the results of the control group with the results of patients with rhinitis, as well as taking into account the criteria presented in the table (Table 1), two groups of patients were distinguished: 10 people with a similar cytological picture indicating allergic rhinitis (AR) and 10 people whose cytological changes suggested nonallergic/vasomotor rhinitis (NAR/VMR) (also known as idiopathic). The atopic nature of rhinitis in AR patients was confirmed by a positive response to house dust mite allergens and common allergens contained in plant pollen in a skin prick test (SPT) and the Polycheck immunoenzymatic test for the presence of allergen-specific IgE (Biocheck GmbH, Münster, Germany). In contrast, patients with NAR/VMR showed a lack of positive response in the above-mentioned studies.

Patients with defined rhinitis qualified for treatment with azelastine hydrochloride spray, which was applied intranasally in the morning and evening (equivalent to 0.56 mg of the compound per day). After 4 weeks of treatment, swabs were again taken from patients.

### 2.2. Methodology of Performing Swabs and Their Evaluation

From each patient, two swabs were taken from the surface of the nasal mucosa using a sterile cytological brush (Jiangsu Yada Technology Group Co, Ltd., Yangzhou, China), precisely from the middle part of the inferior nasal turbinate, which tends to have a significant number of cells and a normal ratio of ciliary cells to goblet cells [22]. A correct swab (cell-rich material suitable for microscopic analysis) can be obtained by rotating the brush with moderate pressure on the sampling surface to collect all the cells, i.e., those building the proper nasal epithelium and inflammatory cells—present in large numbers in pathological conditions. The material was then spread manually into a thin layer over 2/3 of the surface of the degreased slide in a single movement parallel to its edge (avoiding repeated application of the material in the same place). The obtained swabs were stained using two methods: Papanicolaou for the differentiation of epithelial cells (columnar cells, goblet cells, squamous cells, basal cells) and May–Grünwald–Giemsa (MGG) for the detection of inflammatory cells, as well as bacteria, spores, and fungal hyphae [21,23]. The preparations were analyzed using a Nikon ECLIPSE 80i light microscope (Nikon Instruments) with a Nikon Nis Elements D digital image analysis system at ×400, after prior evaluation of slide quality at a lower magnification (×100). In the case of diagnostic doubt, the cells were examined under immersion (×1000). In order to detect all changes significant for diagnostic purposes, the entire surfaces of randomly selected microscope fields of view were carefully read (after previously identifying fields with uniform cellular distribution at low magnification). All possible cytotypes of the nasal mucosa were analyzed in each field of view. The presence of neutrophils, eosinophils, basophils, lymphocytes, monocytes, and mast cells was assessed. However, among the epithelial cells, both cells of the ciliated pseudostratified columnar epithelium (columnar cells, goblet cells, basal cells) and cells of the stratified squamous epithelium (superficial and cells originating from deeper layers of the epithelium) were differentiated. During the morphological assessment, special attention was focused on epithelial cells, and the analyzed changes concerned the cell nucleus (change in size, karyorrhexis, bi- and multinucleated forms, changes typical of apoptosis) and the cytoplasm (change in staining, presence of vacuoles). In addition, a characteristic area above the nucleus called the hyperchromatic supranuclear stria (SNS) was assessed in the ciliary cells, as well as the condition of the ciliary apparatus. It should be mentioned that the method used to collect samples for cytological tests was originally designed for cilia study due to its low invasiveness and high accuracy [24]. The total number of cells counted once per slide (per patient) was 500. Only the presence of ciliary cells with a hyperchromatic area (SNS^+^ cells) was verified on 100 ciliary cells. In order to obtain the most reliable results in each preparation, cells were counted in triplicate, and the final result for each analyzed feature was the average value. The results of the analysis were presented in cytograms and photos.

### 2.3. Statistical Analysis

Statistical analysis of the study results was performed using one-way analysis of variance (ANOVA) with multiple post-hoc comparisons using Tukey’s test. Differences were considered statistically significant at *p* < 0.05. Statistica 13.3 software (StatSoft, Cracow, Poland) was used for data analysis.

## 3. Results

### 3.1. Analysis of Nasal Mucosal Cells of Patients with Rhinitis Symptoms Based on Swabs Taken before Treatment: Evaluation in Relation to the Control Group

#### 3.1.1. Normal Cytogram of the Nasal Mucosa

**Figure 1:**
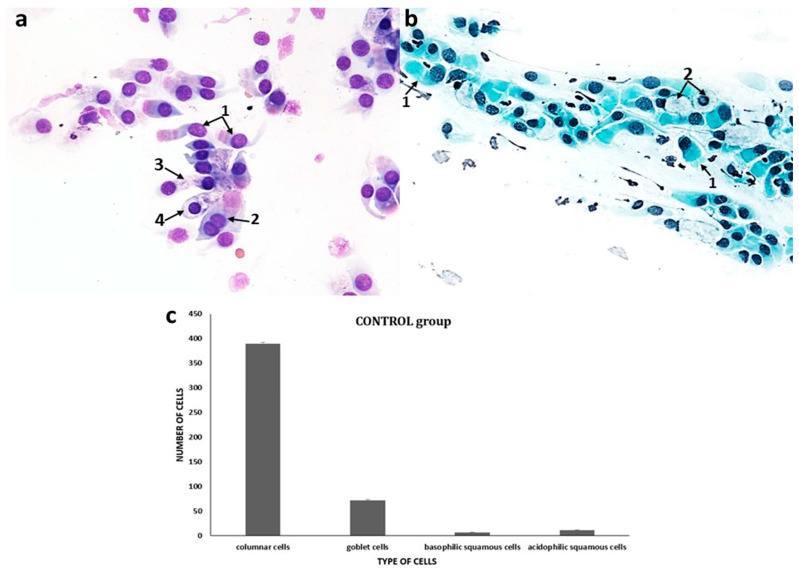
The cytology of normal nasal epithelium under light microscopy (×400): (**a**) MGG staining: 1—columnar cells with normal ciliary apparatus and visible hyperchromatic supranuclear striae (SNS^+^ cells), 2—binucleated columnar cell, 3—columnar cell with cytoplasm vacuolization, 4—perinuclear halo in a columnar cell. (**b**) Papanicolaou staining: 1—columnar cells with normal ciliary apparatus and evident SNS (SNS^+^ cells), 2—goblet cell. (**c**) Distribution of nasal mucosa cells in a normal cytogram.

Cytological study of the control group showed a clear dominance of cells of the pseudostratified ciliated columnar epithelium, i.e., 72 goblet cells and 410 columnar cells remaining with a normal MUC/CIL ratio of 1:6 (Figure 1c). Ciliated cells were identified by their characteristic cylindrical shape and the presence of cilia (Figure 1a,b). In the ciliary cells, a perinuclear halo in the form of a clear area around the nucleus was present (Figure 1a), as well as a normal ciliary apparatus and a hyperchromatic supranuclear stria, which has a red-fuchsia shade in May–Grünwald–Giemsa staining (MGG) (Figure 1a), while in Papanicolaou staining, the SNS is visible as a narrow lucency over the cell nucleus (Figure 1b). The percentage of SNS-positive cells was 89.7% (Figure 12d and Figure 13d). Single columnar cells showed features of vacuolization in the cytoplasm (four cells), while no apoptotic cells were shown (Figure 12c and Figure 13c).

#### 3.1.2. Cytological Profile of Allergic Rhinitis (AR)

**Figure 2:**
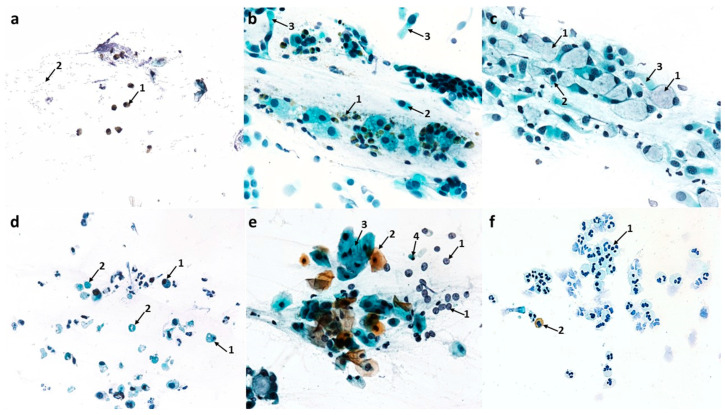
The cytology of the nasal epithelium of patients with allergic rhinitis (AR) before azelastine treatment under light microscopy (×400): Papanicolaou staining: (**a**) 1—eosinophils, 2—bacteria. (**b**) 1—degranulated eosinophils, 2—columnar cells with absence of ciliary apparatus (SNS^−^ cells), 3—columnar cells with rarefaction of ciliary apparatus (SNS^−^ cells). (**c**) 1—giant goblet cells filled with mucus, 2—columnar cells with absence of ciliary apparatus (SNS^−^ cells), 3—columnar cells with rarefaction of ciliary apparatus (SNS^−^ cells). (**d**) 1—apoptotic columnar cells, 2—Creola bodies. (**e**) 1—free nuclei of columnar cells, 2—acidophilic cells of the stratified squamous epithelium, 3—vacuolated basophilic cells of squamous epithelium, 4—apoptotic columnar cell. (**f**) 1—neutrophils, 2—eosinophil.

**Figure 3:**
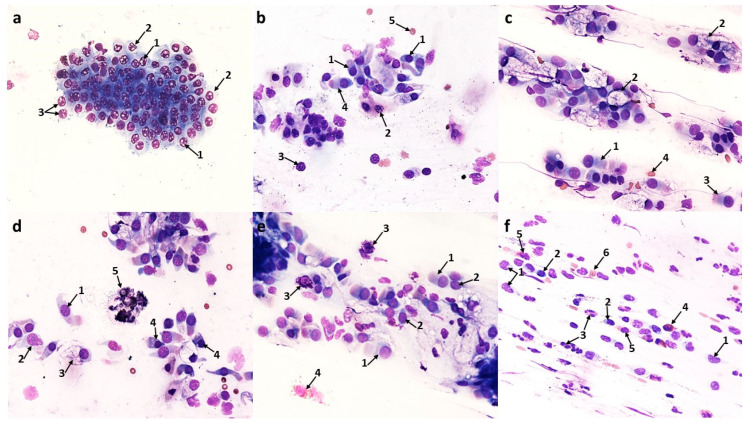
The cytology of the nasal epithelium of patients with allergic rhinitis (AR) before azelastine treatment under light microscopy (×400): MGG staining: (**a**) 1—nuclear vacuolization in columnar cells, 2—blurring of boundaries and atrophy of cytoplasm in columnar cells, 3—free nuclei of columnar cells. (**b**) 1—columnar cells with absence of ciliary apparatus (SNS^−^ cells), 2—granular breakdown of the nucleus (karyorrhexis) in a squamous cell, 3—free nucleus of a columnar cell, 4—columnar cell with normal ciliary apparatus and visible supranuclear stria (SNS^+^ cell), 5—erythrocytes. (**c**) 1—columnar cell with rarefaction of ciliary apparatus (SNS^−^ cells), 2—giant goblet cells filled with mucus, 3—columnar cell with normal ciliary apparatus, 4—erythrocytes. (**d**) 1—columnar cells with rarefaction of ciliary apparatus (SNS^−^ cells), 2—enlarged nucleolus in columnar cell, 3—goblet cell, 4—columnar cells with absence of ciliary apparatus (SNS^−^ cells), 5—inhaled dandelion pollen grain. (**e**) 1—columnar cells with blurred boundaries of cytoplasm and rarefaction of the ciliary apparatus (SNS^−^ cells), 2—columnar cells with absence of ciliary apparatus (SNS^−^ cells), 3—degranulated mast cells, 4—degranulated eosinophil. (**f**) 1—macrophages, 2—lymphocytes, 3—neutrophils, 4—eosinophils, 5—degranulated eosinophils, 6—erythrocytes.

Compared to the control image, swabs from patients with symptoms of allergic rhinitis showed numerous changes in the ciliated pseudostratified columnar epithelium, i.e., in the form of a significant reduction in the number of columnar cells to 72 (*p* ≤ 0.0001) and goblet cells to 24.6 (*p* ≤ 0.0001), and at the same time, a disturbance in the MUC/CIL ratio, which was about 1:3 (Figure 12a). Columnar cells showed very significant morphological differences, such as atrophy of the cytoplasm or blurred contour, damage to the ciliary apparatus (absence or rarefaction), and the absence of hyperchromatic supranuclear stria—SNS (Figure 2b,c and Figure 3b–e). The percentage of cells with a visible hyperchromatic area (SNS^+^ cells) decreased highly statistically to 17.5% (*p* ≤ 0.0001) compared to the control value (Figure 12d). The image clearly showed the presence of very numerous columnar cells in the stage of apoptosis—approximately 46 cells/72 columnar cells in total (Figure 12c), which, compared to the control, was a highly statistically significant result (*p* ≤ 0.0001). Apoptosis was confirmed by changes such as cell shrinkage and increased basophilic cytoplasm, pyknotic and condensed cell nucleus, as well as the presence of Creola bodies (Figure 2d,e). In addition, one could see quite numerous free nuclei of these cells (Figure 2e and Figure 3a,b), enlarged nucleoli (Figure 3d), and nuclear vacuolization (Figure 3a). Goblet cells showed signs of swelling (Figure 2c and Figure 3c).

Significant changes also concerned the cells of the stratified squamous epithelium, as we observed an increase in the number of superficial cells with acidophilic cytoplasm to 39.8 (*p* ≤ 0.0001) and a more than 10-fold increase in the number of basophilic cells originating from the deeper layers of the epithelium to 77.2 (*p* ≤ 0.0001) (Figure 2e and Figure 12a). Some of the squamous cells were characterized by the presence of nuclei with features of granular breakdown (karyorrhexis) (Figure 3b) and the presence of vacuolization changes in the cytoplasm (Figure 2e). Cells with vacuolization changes constituted a statistically significant number (16.7) (*p* ≤ 0.0001) compared to the control (Figure 12c).

In the analyzed cytogram, the dominant change was a highly statistically significant increase in the number of neutrophils to 229 (*p* ≤ 0.0001) (Figure 2f, Figure 3f and Figure 12a), which constituted approximately 46% of all cells analyzed. Also present among the inflammatory cells were eosinophils, including those with degranulation features (Figure 2a,b,f and Figure 3e,f), whose number increased statistically significantly to 55 (*p* ≤ 0.0001) compared to the control (Figure 12a), while basophils, lymphocytes, and monocytes were less abundant (Figure 3f and Figure 12a). Also noteworthy were degranulated mast cells (Figure 3e) and macrophages (Figure 3f). Other changes observed were bacteria, which were present in small individual clusters loosely scattered over the surface of the preparation (Figure 2a), as well as erythrocytes (Figure 3b,c,f) and dandelion pollen grains (Figure 3d).

#### 3.1.3. Cytological Profile of Nonallergic/Vasomotor Rhinitis (NAR/VMR)

**Figure 4:**
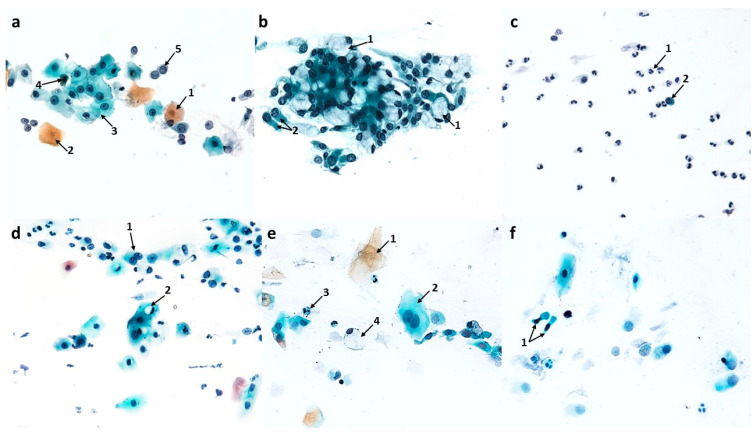
The cytology of the nasal epithelium of patients with nonallergic/vasomotor rhinitis (NAR/VMR) before azelastine treatment under light microscopy (×400). Papanicolaou staining: (**a**) 1—acidophilic squamous cells (superficial cells), 2—acidophilic cell with a nuclear shadow, 3—basophilic squamous cells, 4—apoptotic squamous cell, 5—free columnar cell nuclei. (**b**) 1—swollen goblet cells, which outnumber ciliated cells (muciparous metaplasia), 2—columnar cells. (**c**) 1—neutrophils, 2—apoptotic columnar cell with visible nuclear fragmentation. (**d**) 1—columnar cells with blurred boundaries of cytoplasm and rarefaction of the ciliary apparatus (SNS^−^ cells), 2—vacuolated basophilic cells of squamous epithelium. (**e**) 1—acidophilic squamous cells, 2—binucleated basophilic cell of squamous epithelium, 3—neutrophil, 4—goblet cell. (**f**) 1—apoptotic columnar cells.

**Figure 5:**
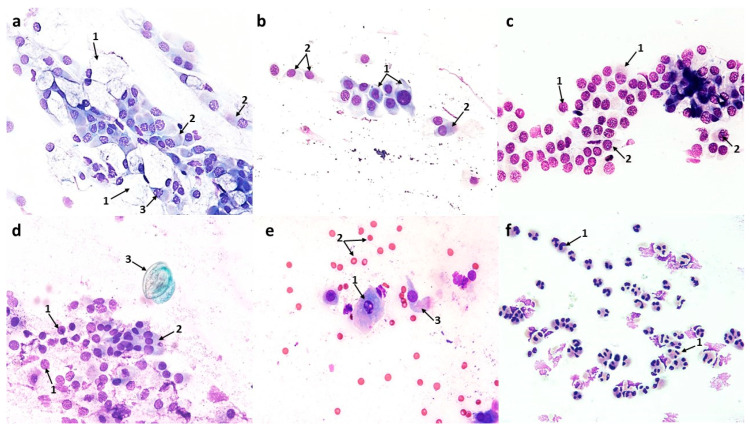
The cytology of the nasal epithelium of patients with nonallergic/vasomotor rhinitis (NAR/VMR) before azelastine treatment under light microscopy (×400): MGG staining: (**a**) 1—increased number of swollen goblet cells filled with mucus (muciparous metaplasia), 2—columnar cells with rarefaction of ciliary apparatus (SNS^−^ cells), 3—nuclear vacuolization in a columnar cell. (**b**) 1—metaplastic cells, 2—columnar cells with rarefaction of ciliary apparatus (SNS^−^ cells). (**c**) 1—blurring of cell boundaries, atrophy of cytoplasm, and absence of ciliary apparatus in columnar cells (SNS^−^ cells), 2—enlarged nucleoli in columnar cells. (**d**) 1—atrophy of cytoplasm in columnar cells, 2—columnar cells with rarefaction of ciliary apparatus (SNS^−^ cells), 3—inhaled pine pollen grain. (**e**) 1—nuclear vacuolization in squamous cell, 2—erythrocytes, 3—columnar cell with rarefaction of ciliary apparatus (SNS^−^ cells). (**f**) 1—neutrophil infiltration.

Compared to the control, the dominant change was a highly statistically significant increase in the number of goblet cells to 119.8 (*p* ≤ 0.0001) with a simultaneous reduction in the number of columnar cells to 270.1 (*p* ≤ 0.0001) (Figure 13a), which indicated a significant disturbance in the proportion of these cells in the ciliated epithelium, and the MUC/CIL ratio was 1:2. The goblet cells showed signs of severe swelling due to being filled with a large amount of mucus (Figure 4b and Figure 5a). Columnar cells were characterized by blurred cell boundaries as well as atrophy of the cytoplasm (Figure 4d and Figure 5c,d), hence free cell nuclei were present in the swabs (Figure 4a). Other pathological features were an enlarged nucleolus (Figure 5c), nuclear vacuolization (Figure 5a), and damage to the ciliary apparatus (absence or rarefaction of cilia) (Figure 4d and Figure 5a–e). SNS-positive cells constituted only approximately 10%, which was a statistically significant change compared to the control (*p* ≤ 0.0001) (Figure 13d). The presence of metaplastic cells was also demonstrated (Figure 5b). Only single columnar cells had apoptotic features, i.e., shrunken and basophilic cytoplasm, pyknotic and condensed cell nucleus (also with fragmentation features) (Figure 4c,f).

The images analyzed showed numerous changes in the stratified squamous epithelium. There was an increase in the number of superficial acidophilic cells, also with a prominent nuclear shadow, to 35.2 (*p* ≤ 0.0001) and basophilic cells originating in the deeper layers of the epithelium to 29 (*p* ≤ 0.0001) (Figure 4a and Figure 13a). Numerous degenerative changes in these cells, such as vacuolization in the cytoplasm (Figure 4d), nuclear vacuolization (Figure 5e), binucleated cells (Figure 4e), and cells in apoptosis (Figure 4a), were also demonstrated. Overall, the number of epithelial cells with vacuolization and apoptotic changes was statistically insignificant and amounted to 6.4 and 3.3, respectively (Figure 13c).

Cytological analysis showed a highly statistically significant increase in neutrophils to 45.9 (*p* ≤ 0.0001) (Figure 4c, Figure 5f and Figure 13a), as well as the presence of quite numerous erythrocytes loosely scattered on the surface of the preparation (Figure 5e) and pine pollen grains (Figure 5d).

### 3.2. Analysis of Nasal Mucosal Cells of Patients with Rhinitis Symptoms Based on Swabs Taken after Treatment with Azelastine Hydrochloride: Evaluation in Relation to the State before Treatment

#### 3.2.1. Changes Induced in Nasal Mucosal Cells of Patients with Allergic Rhinitis (AR)

**Figure 6:**
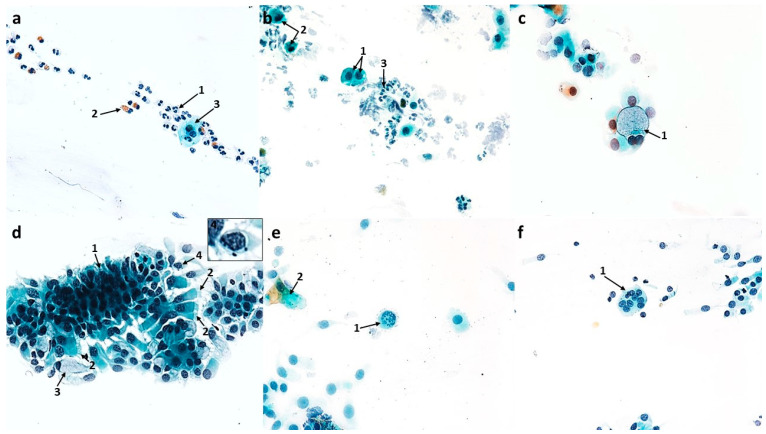
The cytology of the nasal epithelium of patients with allergic rhinitis (AR) after azelastine treatment under light microscopy (×400): Papanicolaou staining: (**a**) 1—neutrophils, 2—intact eosinophils, 3—squamous cell. (**b**) 1—basal cells with cytoplasm vacuolization, 2—apoptotic basophilic cells of squamous epithelium, 3—neutrophils. (**c**) 1—binucleated goblet cell. (**d**) 1—cluster of columnar epithelium cells, 2—columnar cells with normal ciliary apparatus (SNS^+^ cells), 3—goblet cell, 4—perinuclear halo in columnar cell. (**e**) 1—cell at the stage of mitotic division, 2—squamous cells. (**f**) 1—multinucleated columnar cell.

**Figure 7:**
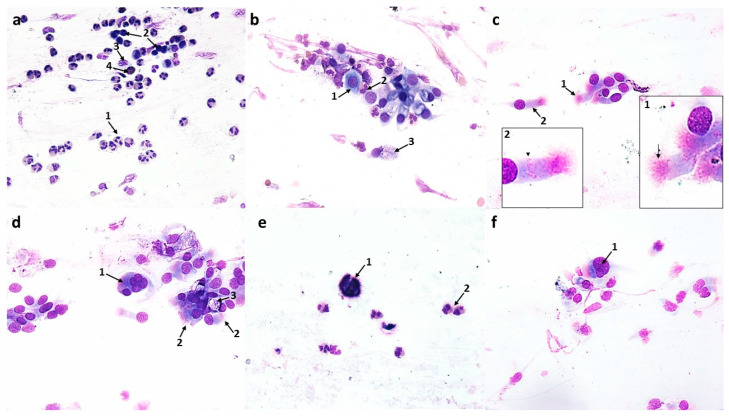
The cytology of the nasal epithelium of patients with allergic rhinitis (AR) after azelastine treatment under light microscopy (×400): MGG staining: (**a**) 1—neutrophils, 2—lymphocytes, 3—macrophages, 4—intact eosinophil. (**b**) 1—basal cell, 2—intact eosinophil, 3—goblet cells. (**c**) 1—columnar cells with normal ciliary apparatus, 2—columnar cells with distinct hyperchromatic supranuclear striae (SNS^+^ cells). (**d**) 1—multinucleated columnar cell, 2—columnar cells with normal ciliary apparatus and distinct SNS (SNS^+^ cells), 3—goblet cell. (**e**) 1— inhaled dandelion pollen grain, 2—neutrophils. (**f**) 1—swollen cell nucleus in a columnar cell.

Compared to the pre-treatment condition, changes were observed in the population of nasal mucosal cells, definitely in favor of the cells of the ciliated pseudostratified columnar epithelium. There was a statistically significant increase in the number of columnar cells to 290.9 (*p* ≤ 0.0001), which formed larger clusters and had clearly stained and well-contoured cytoplasm (Figure 6d and Figure 12b) and an increase in the number of goblet cells to 47.5 (*p* ≤ 0.0001), which did not show signs of swelling (Figure 6d, Figure 7b,d and Figure 12b). At the same time, there was a highly statistically significant increase in the percentage of cells with visible hyperchromatic supranuclear stria and normal ciliary apparatus to 75% (*p* ≤ 0.0001) (Figure 6d, Figure 7c,d and Figure 12d). Additionally, the following cells were present: basal (Figure 6b and Figure 7b), binucleated (Figure 6c), multinucleated (Figure 6f and Figure 7d), with a swollen cell nucleus (Figure 7f), and at the stage of cell division (Figure 6e).

A significant qualitative change observed in columnar cells was the intensification of vacuolization changes in the cytoplasm (75.2 cells), which was a highly statistically significant result (*p* ≤ 0.0001) (Figure 12c). The cells contained a single large vacuole (Figure 10d) or the vacuoles were so numerous that the cells had a transparent cytoplasm (Figure 10a,c). Attention was drawn to the presence of vacuoles with contents clearly visible in their lumen, which occurred in the cytoplasm of columnar cells (Figure 10a) and squamous cells (Figure 10b). It should be noted that among the vacuolated cells visible in MGG staining, the presence of forms with a significantly widened area with regular brightening of the cytoplasm at the site of the supranuclear stria (Figure 10e,f) was demonstrated. In columnar cells, there was a characteristic clear area around the nucleus, the so-called perinuclear halo (Figure 10c), and no changes typical of apoptosis were detected.

At the same time, the number of stratified squamous epithelium cells decreased (Figure 12b), i.e., basophilic cells to 10.9 (*p* ≤ 0.0001) and acidophilic cells to 22.4 (*p* ≤ 0.0001). Cells with apoptotic changes could be observed (Figure 6b), the number of which decreased highly statistically significantly to 4.5 (*p* ≤ 0.0001) (Figure 12c).

The analyzed images showed the presence of inflammatory cells, such as neutrophils and eosinophils (without signs of degranulation) (Figure 6a and Figure 7a); however, their number was reduced to 106.9 and 19.9, respectively (Figure 12b), which were highly statistically significant results (*p* ≤0.0001). There were no significant changes in the number of basophils, lymphocytes, or monocytes (Figure 12b). Dandelion pollen grains were often present in the swabs (Figure 7e).

#### 3.2.2. Changes Induced in Nasal Mucosal Cells of Patients with Nonallergic/Vasomotor Rhinitis (NAR/VMR)

**Figure 8:**
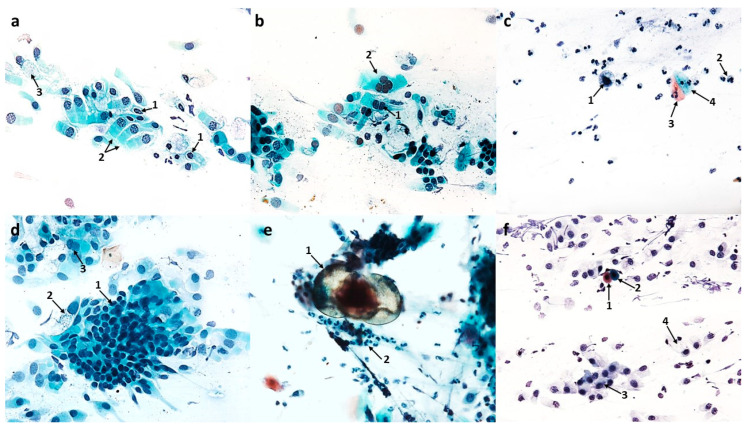
The cytology of the nasal epithelium of patients with nonallergic/vasomotor rhinitis (NAR/VMR) after azelastine treatment under light microscopy (×400). Papanicolaou staining: (**a**) 1—columnar cells with perinuclear halo, 2—columnar cells with normal ciliary apparatus and distinct hyperchromatic supranuclear stria (SNS^+^ cells), 3—goblet cells. (**b**) 1—binucleated columnar cell, 2—multinucleated columnar cell. (**c**) 1—basal cell with cytoplasmic vacuolization, 2—neutrophils, 3—acidophilic cell of squamous epithelium, 4—basophilic cell of squamous epithelium. (**d**) 1—cluster of columnar epithelial cells, 2—goblet cell, 3—binucleated columnar cell. (**e**) 1—inhaled pine pollen grain, 2—neutrophils. (**f**) 1—apoptotic acidophilic cell of squamous epithelium, 2—apoptotic basophilic cell of squamous epithelium, 3—columnar cells, 4—goblet cell.

**Figure 9:**
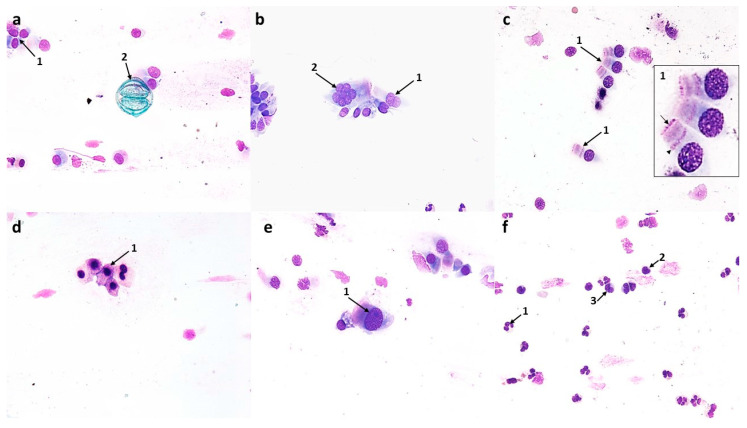
The cytology of the nasal epithelium of patients with nonallergic/vasomotor rhinitis (NAR/VMR) after azelastine treatment under light microscopy (×400): MGG staining: (**a**) 1—columnar cells, 2—inhaled pine pollen grain. (**b**) 1—binucleated columnar cell, 2—multinucleated columnar cell. (**c**) 1—columnar cells with normal ciliary apparatus and distinct hyperchromatic supranuclear stria (SNS^+^ cells). (**d**) 1—apoptotic cells of squamous epithelium. (**e**) 1—swollen nucleus in a columnar cell. (**f**) 1—neutrophils, 2—lymphocytes, 3—monocytes.

Cytological analysis of the nasal mucosa in the studied patients after completion of treatment showed numerous changes. The ciliated epithelium was significantly modified, i.e., the number of goblet cells was reduced to 52.6 (*p* ≤ 0.0001), while the number of columnar cells increased to 340.5 (*p* ≤ 0.0001), which improved the MUC/CIL ratio (1:6) (Figure 13b). Columnar cells showed morphological differentiation into bi- and multinucleated cells (Figure 8b and Figure 9b), cells with swollen nuclei (Figure 9e), and basal cells (Figure 8c). Moreover, columnar cells formed larger clusters (Figure 8d), had a normal ciliary apparatus and a clearly visible hyperchromatic supranuclear stria (Figure 8a and Figure 9c). The percentage of SNS-positive cells increased statistically significantly to 51.4% (*p* ≤ 0.0001) (Figure 13d). Numerous vacuolization changes visible in the cytoplasm as unstained spaces were very characteristic of the columnar cells, resulting in a strong brightening of the cytoplasm (Figure 11e,f). Vacuoles with dark-colored contents were identified in some cells (Figure 11c). The number of vacuolated cells increased statistically significantly to 109 (*p* ≤ 0.0001) (Figure 13c). Noteworthy is the significantly widened area with regular brightening of the cytoplasm precisely at the site of the hyperchromatic stria (Figure 11a), as well as the presence of numerous vacuoles in this area (Figure 11b,d). There was a perinuclear halo in columnar cells (Figure 11a,c). The presence of columnar cells with apoptotic changes was not detected.

Statistically significant changes were noted in the cells of the stratified squamous epithelium (Figure 8c and Figure 13b): a reduction in the number of basophilic cells and acidophilic cells to 7.9 and 13.4, respectively, which, in both cases, was a highly statistically significant result (*p* ≤ 0.0001). Morphological analysis showed the presence of squamous cells in the apoptotic stage (Figure 8f and Figure 9d), the number of which increased statistically significantly to 41.5 (*p* ≤ 0.0001) (Figure 13c). Some cells showed the presence of vacuolization changes in the cytoplasm (Figure 11b).

In the analyzed preparations, inflammatory cells were present, such as neutrophils, the number of which increased statistically significantly to 83.2 (*p* ≤ 0.0001) (Figure 8c, Figure 9f and Figure 13b), and lymphocytes and monocytes, which occurred in lower but statistically significant numbers (Figure 9f and Figure 13b). Pine pollen grains were identified in the swabs (Figure 8e and Figure 9a).

**Figure 10:**
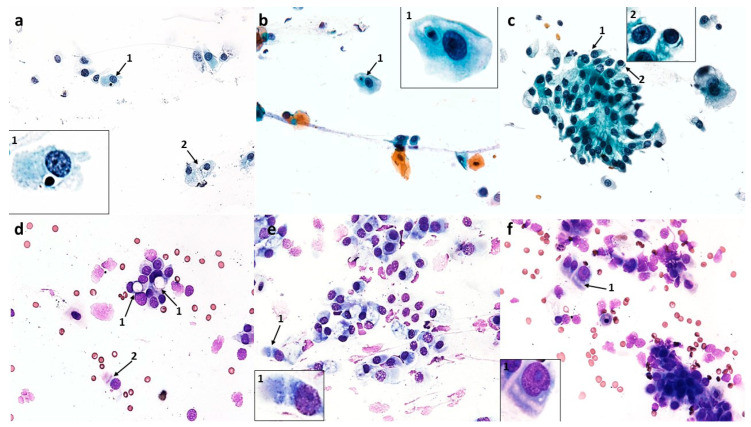
Vacuolization changes in epithelial cells in patients with allergic rhinitis (AR) induced by the action of azelastine (light microscope observation, ×400). Papanicolaou (**a**–**c**) and MGG staining (**d**–**f**). (**a**) 1—columnar cell with a vacuole, in the light of which dark-colored contents are visible, 2—vacuolated columnar cells with transparent cytoplasm. (**b**) 1—basophilic squamous cell with a vacuole with distinctly marked contents. (**c**) 1—cluster of columnar cells with intense cytoplasmic vacuolization, 2—perinuclear halo in columnar cell. (**d**) 1—columnar cells with giant vacuoles, 2—columnar cell with distinct supranuclear stria (SNS^+^ cell). (**e**,**f**) 1—columnar cells with a significantly widened area with regular brightening of the cytoplasm at the site of SNS.

**Figure 11:**
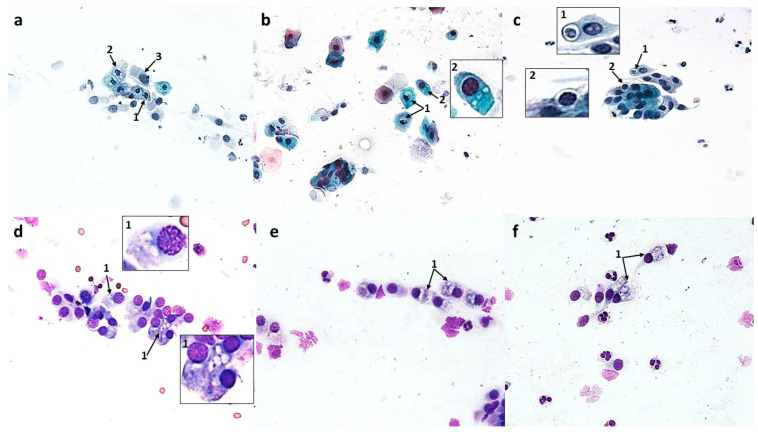
Vacuolization changes in epithelial cells in patients with nonallergic/vasomotor rhinitis (NAR/VMR) induced by the action of azelastine (light microscope observation, ×400). Papanicolaou (**a**–**c**) and MGG staining (**d**–**f**). (**a**) 1—perinuclear halo in columnar cells, 2—columnar cells with intense cytoplasmic vacuolization, 3—columnar cell with a significantly widened area with regular brightening of the cytoplasm precisely at the site of the hyperchromatic stria. (**b**) 1—basophilic cells with vacuolization changes, 2—columnar cell with vacuoles in the SNS area. (**c**) 1—columnar cell with a large vacuole, in the light of which darkly colored contents are visible, 2—perinuclear halo in columnar cell. (**d**) 1—columnar cells with numerous vacuoles in the SNS area. (**e**,**f**) 1—strongly brightened columnar cells with numerous small vacuoles in the cytoplasm.

**Figure 12:**
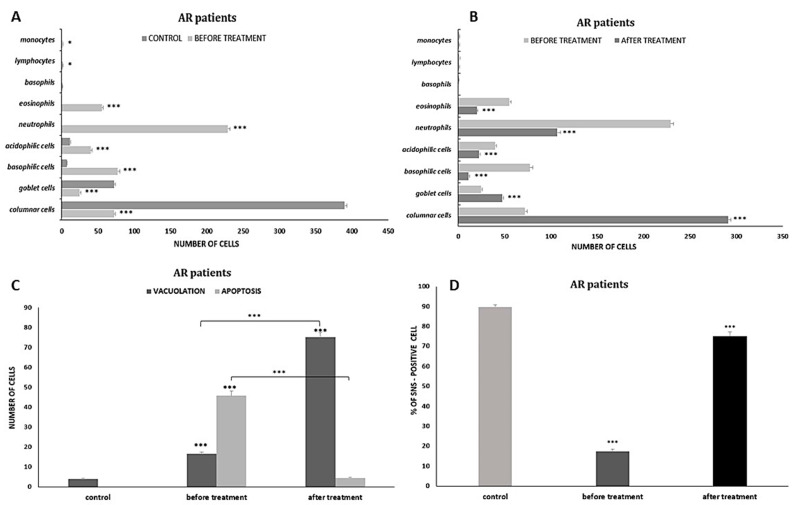
Quantitative analysis of changes demonstrated in the nasal mucosa of patients with allergic rhinitis (AR) before and after azelastine therapy. (**A**) Distribution of epithelial and inflammatory cells in swabs before treatment; *** *p* < 0.001, * *p* < 0.05 relative to control. (**B**) Distribution of epithelial and inflammatory cells in swabs after treatment; *** *p* < 0.001 relative to pre-treatment condition. (**C**) Number of vacuolated cells and apoptotic cells before treatment (*** *p* < 0.001 relative to control) and after completion of therapy (*** *p* < 0.001 compared to the results before treatment). (**D**) Percentage of SNS-positive cells before treatment (*** *p* < 0.001 relative to control) and after treatment (*** *p* < 0.001 relative to pre-treatment condition).

**Figure 13:**
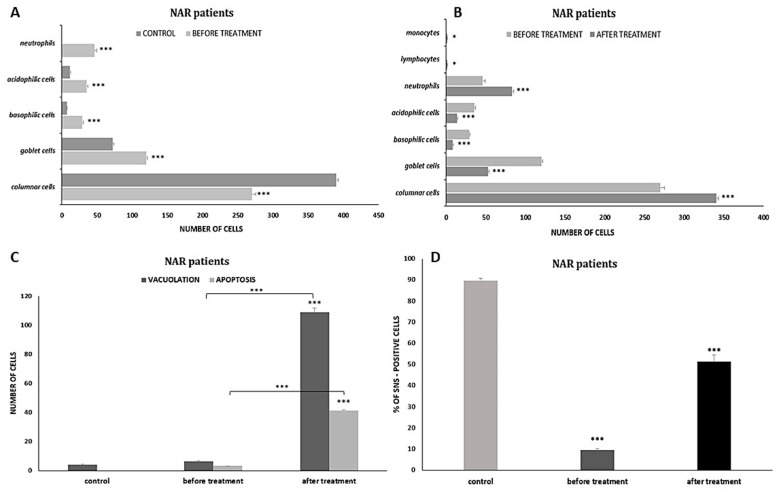
Quantitative analysis of changes demonstrated in the nasal mucosa of patients with nonallergic/vasomotor rhinitis (NAR/VMR) before and after azelastine therapy. (**A**) Distribution of epithelial and inflammatory cells in swabs before treatment; *** *p* < 0.001 relative to control. (**B**) Distribution of epithelial and inflammatory cells in swabs after treatment; *** *p* < 0.001, * *p* < 0.05 relative to pre-treatment condition. (**C**) Number of vacuolated cells and apoptotic cells before treatment (*** *p* < 0.001 relative to control) and after completion of therapy (*** *p* < 0.001 compared to the results before treatment). (**D**) Percentage of SNS-positive cells before treatment (*** *p* < 0.001 relative to control) and after treatment (*** *p* < 0.001 relative to pre-treatment condition).

## 4. Discussion

The proper treatment of particular types of rhinitis depends on the accurate and personalized diagnosis of patients [4]. The relative frequency and clinical course of allergic rhinitis (AR) and nonallergic rhinitis (NAR) are similar, making their differentiation difficult [25]. Furthermore, the term ‘nonallergic rhinitis’ represents a heterogeneous group of conditions with different triggers and different pathophysiologies [25,26], which cause similar nasal symptoms and are therefore often underdiagnosed [21]. In turn, AR may show different forms and severity of inflammation depending on allergic sensitization and possibly the season [21]. Differentiating between allergic and nonallergic rhinitis is extremely important to better classify the patient’s pathology and thus determine the appropriate form of treatment depending on the dominant cellular population [27].

Nasal exfoliative cytology is a simple tool for assessing normal and pathological aspects of the nasal mucosa by identifying and counting individual cytotypes and studying their morphology [21,28,29,30]. Cytological findings are generally used to identify the mechanisms playing a fundamental role in the development of rhinitis and to link the cytological picture to nasal pathology [26]. The available literature contains information on the role of cytodiagnostics in the differentiation of AR and NAR, but most studies concern the identification of eosinophils in order to narrow the differential diagnosis with other types of rhinitis [31,32,33,34,35]. However, there is a lack of detailed quantitative data regarding other cytotypes of the nasal mucosa, especially resident cells, as well as data on morphological changes in cells in both forms of rhinitis, which may be very important for the diagnosis and treatment of these diseases.

Most importantly, nasal cytology is an objective test recommended by ARIA that allows the monitoring of medical treatment in various forms of nasal diseases [26]. It can also be a useful tool for research purposes [21]. In the literature, little attention has been paid to cytological study conducted with a view to learning new mechanisms of action of intranasally administered drugs identified at the cellular level at the site of their direct action—in the nasal mucosa. So far, detailed cytological changes in the nasal mucosa in patients with nonallergic/vasomotor rhinitis and patients with allergic rhinitis have not been described.

Preparations from patients belonging to both analyzed groups had very good cellularity, which allowed for a thorough analysis and obtaining reliable results necessary to establish detailed cytological profiles and, at a later stage of research, to reliably assess the effects of the therapy.

In the swabs of AR group patients before treatment, neutrophils were the predominant cytotype (Figure 2f, Figure 3f and Figure 12a), while eosinophils, often with degranulation features, were present in four times lower numbers (Figure 2a,b and Figure 3e,f). As is commonly known, eosinophils are an important marker of allergic inflammation [36], but the literature lacks precise criteria for identifying nasal eosinophilia. Our results are confirmed by the study of Żyła [37], where the percentage of these cells in swabs of patients with allergic seasonal rhinitis was in the range of 5–10% and was significantly correlated with the presence of clinical symptoms of allergy. According to reports by Mierzejewska [38] and Bartoli [39], eosinophilia in swabs is more sensitive and specific in detecting allergic rhinitis compared to biopsy. Recently, the involvement of neutrophils in the development of allergic inflammation has also been highlighted [40]. Some studies have shown that in the nose of patients with allergic symptoms during the peak pollen season, a significant share of inflammatory cells is attributed to neutrophils, which greatly outnumber eosinophils [41]. Similar to our study, elevated levels of neutrophils relative to eosinophils were independently demonstrated by Ciprandi [42] and Chen [33] in the nasal mucosa as a consequence of continuous low-level exposure to mite allergens (HDM). At the same time, the presence of other inflammatory cells confirmed in our studies, i.e., lymphocytes (Figure 3f), macrophages (Figure 3f), and mast cells (Figure 3e), indicates the severity of the allergic reaction. The severity of allergic rhinitis is associated with varying numbers of cells identified in nasal cytology; especially in moderate and severe AR, there is a significantly increased number of mast cells and lymphocytes [43,44]. The presence of numerous erythrocytes (Figure 3b,c,f), which indicate swelling of the nasal mucosa, also confirm the intense inflammation in the examined swabs.

The release of various mediators from accumulated cells during the late phase of the nasal allergic reaction is responsible for both the symptoms of allergic rhinitis and other pathological changes in the nasal epithelium [44]. Hence, a consistent feature of the swabs of patients with AR was the changes in the ciliated epithelium shown in our study. Columnar cells showed signs of degeneration confirmed by numerous changes in the morphological profile. More than half of the columnar cells had typical apoptotic features (Figure 2d,e and Figure 12c), and the death of these cells by apoptosis was also confirmed by Creola bodies (Figure 2d). These changes correlated with a significant reduction in cells of the ciliated epithelium and a disruption of the MUC/CIL ratio (Figure 12a). Admittedly, goblet cells showed features of swelling (Figure 2c and Figure 3c), but ultimately the features of muciparous metaplasia were not confirmed. In turn, changes demonstrated in living columnar cells include atrophy of the cytoplasm and its unclear contouring (Figure 3a,e), vacuolization changes in the cell nucleus (Figure 3a), enlarged nucleoli (Figure 3d), and the presence of free cell nuclei (Figure 2e and Figure 3a,b), which are typical of reactive degenerative changes, often accompanying disturbed biocenosis [26,45]. Admittedly, we showed the presence of bacteria in the analyzed preparations, but they were only single small clusters (Figure 2a), which should be considered a consequence of the dysfunction of mucociliary transport. On this basis, it was also possible to exclude an infectious agent as the cause of rhinitis in the examined patients. The disturbance of mucociliary clearance was, in turn, the result of damage to the ciliary apparatus in columnar cells, and this was confirmed by the absence of the hyperchromatic supranuclear stria (Figure 2b,c and Figure 3b–e). According to the literature, this hyperchromatic area consists of protein components of the ciliary apparatus. The disappearance of the supranuclear stria is observed in patients with disorders of the nasal mucosa and is an expression of distress phenomena related to the deficiency of ciliary proteins and the loss (amputation) or rarefaction of the ciliary apparatus [46,47]. In our studies, we estimated the number of cells with a distinct SNS (SNS^+^ cells), which was strongly reduced compared to the control (Figure 12d). The ciliary cell is highly differentiated compared to other cytotypes of the respiratory mucosa and is the one most affected by degenerative processes during inflammatory diseases. From the cellular point of view, various stimuli, including allergens, infections, and physicochemical or irritating factors, acting on the nasal mucosa first affect the ciliated cells, causing significant changes in them [46]. A marker of cellular modifications of the nasal epithelium was the presence of very numerous squamous cells at various stages of development (Figure 2e and Figure 12a). Since no metaplastic cell stage was detected in the examined swabs, this may suggest that the appearance of squamous epithelial cells is the result of already existing inflammatory changes. As reported by Myszkowska [48], in patients with allergic rhinitis, squamous epithelial cells may appear in larger numbers due to the reaction of the sensitive nasal mucosa to airborne allergens to which the patient is allergic. Also, the cytoplasmic vacuolization (Figure 2e) and the breakdown of the cell nucleus (karyorrhexis) (Figure 3b) demonstrated in squamous cells are examples of degenerative changes in inflammatory conditions [49,50].

Swabs from patients with NAR before treatment showed numerous changes in the ciliated pseudostratified columnar epithelium. In contrast to AR, a two-fold increase in the number of swollen goblet cells was characteristic here (Figure 4b and Figure 5a), with a simultaneous reduction in columnar cells (Figure 13a). Both columnar and mucus-secreting cells represent the first line of defense localized in the airways [51,52] under certain conditions determining the remodeling of the mucosal epithelium in favor of mucus-secreting cells [53]. The dominant mechanism for the increase in the number of these cells is the conversion of columnar cells into goblet cells, which is called muciparous metaplasia [54]. Both chronic stimulation of the airway with irritants and acute external stimuli can increase the number and size of goblet cells as well as the intracellular amount of mucin [38]. The obtained data confirm vasomotor rhinitis, also called nonallergic non-infectious perennial rhinitis or idiopathic rhinitis [55]. A higher percentage of goblet cells in nonallergic non-infectious rhinitis was also confirmed by Canakcioglu’s team [31], but according to them, this is the only significant difference in the cytological picture between patients with NAR and AR, which in turn contrasts with our findings. Vasomotor rhinitis is characterized by distinct symptoms of nasal obstruction and congestion [56], hence the presence of numerous erythrocytes in the analyzed swabs (Figure 5e). Damage to the ciliated epithelium is also evidenced by squamous metaplasia, which involves the replacement of the columnar epithelium with stratified squamous epithelium [51]; this was also confirmed in the analyzed swabs by the presence of metaplastic cells (Figure 5b) and an increased number of squamous cells (Figure 4a and Figure 13a). Such changes can be explained by the chronic impact of external factors causing hypersensitivity of the nasal mucosa. Similarly, in Myszkowska’s study [48], squamous cell metaplasia was demonstrated in the cytological picture of a patient with nonallergic rhinitis under the influence of volatile irritants such as a fragrance mixture. Analogously to the group with allergic rhinitis, in the examined swabs, columnar cells showed abnormalities typical of pathological conditions, such as blurred cell boundaries and atrophy of the cytoplasm (Figure 5c,d), a free cell nucleus (Figure 4a), or an enlarged nucleolus (Figure 5c). The similarity also concerned the damage to the ciliary apparatus (Figure 4d and Figure 5a–e), which was reflected in a significant reduction in the percentage of SNS-positive cells, slightly lower than in the AR group (Figure 13d). As reported by Gelardi [46], the percentage of cells with a visible hyperchromatic stria decreased in cytological preparations of nasal mucosa affected by inflammatory or degenerative processes, depending on the severity of the pathological picture. Swabs from NAR patients did not show an increased number of apoptotic columnar cells (Figure 4c,f and Figure 13c), which is in contrast to the AR group. In turn, the slight apoptotic and vacuolization changes concerned squamous cells (Figure 4a,d, Figure 5e and Figure 13c) and were a manifestation of degeneration characteristic of inflammation [54], similar to the presence of binucleated forms observed in these cells (Figure 4e).

In the examined material, the pathological cell population was quite numerous in neutrophils (Figure 4c, Figure 5f and Figure 13a). Similar results were obtained by Gelardi [54], showing that the rhinological cytogram of a child with acute or chronic nonallergic rhinitis, compared to healthy people, may present a higher percentage of neutrophils and an inversion of the ratio of ciliated and goblet cells in favor of the latter, which is also confirmation of the previously discussed muciparous metaplasia. The absence of other inflammatory cells in the analyzed swabs is supported by the literature, because according to previous studies, patients diagnosed with chronic VMR and symptoms of chronic rhinitis lasting one year or more are characterized by negative nasal cytology for eosinophils [57]. Knowledge of the pathophysiology of NAR subtypes and their differentiation is quite extensive, but the literature lacks detailed information on the cytological picture of VMR, so the results of our study complement the data in this area.

Cytological changes in swabs of AR patients after azelastine treatment showed a reduction of the pathological condition in the nasal mucosa, which was confirmed by the lower density of the preparations. In our study, azelastine significantly reduced the number of eosinophils in a group of patients with allergic rhinitis, while the number of neutrophils was reduced by half (Figure 6a, Figure 7a and Figure 12b). At the same time, increased numbers of monocytes, lymphocytes, and basophils were not observed (Figure 12b). Although the complete disappearance of inflammatory cells in cytological samples was not demonstrated, their significant reduction was observed. Single dandelion pollen grains, which were identified in the swabs of the examined patients (Figure 7a), have a low clinical significance for the induction of inhalant allergy according to the literature, although in some cases they may intensify allergy symptoms [2]. However, taking into account all the results obtained, especially the lack of degranulated eosinophils and mast cells, which were a constant feature of cytological images before treatment, we did not confirm an exacerbation of the allergic response. According to the literature, a significant reduction in inflammatory cell infiltration during treatment may indicate a resolution of the pathological condition [31,58]. Our results are consistent with previous reports that the anti-allergic/anti-inflammatory effects of azelastine are related to, among other things, effects on cellular elements of the allergic phase of inflammation [59]. Two studies by Ciprandi [59,60] showed that intranasally administered azelastine significantly reduces neutrophilic and eosinophilic infiltrates during both the early and later phases of the allergic response compared to the placebo group.

Differential effects of azelastine were noted in relation to the ciliated pseudostratified columnar epithelium. An important aspect is the restoration of mucosal homeostasis expressed on several levels. Columnar cells did not show any degenerative changes typical of the inflammatory process, such as free cell nuclei, vacuolization changes in the cell nucleus, or an enlarged nucleolus, so in this context we can also conclude that azelastine exerts an anti-inflammatory effect. However, significant changes were identified, such as the presence of numerous multinucleated cells (Figure 6f and Figure 7d) and binucleated cells (Figure 6c), which indicate the gradual regeneration of the ciliated epithelium [26,49,50] The renewal of the epithelium was also supported by the basal cells present in the swab (Figure 6b and Figure 7b), which are stem cells for columnar and goblet cells [20]. Damage to the ciliated epithelium caused by inflammatory mediators may require increased epithelial cell turnover to repair this damage [61]. The stimulating effect of azelastine on columnar cells was expressed by the presence of division figures (Figure 6e), the occurrence of these cells in large clusters (Figure 6d), and the normalization of the MUC/CIL ratio in favor of columnar cells (Figure 12b). The improvement in the condition of the ciliated epithelium was also confirmed by the correct morphological profile of columnar cells, as they were characterized by a clearly stained and well-contoured cytoplasm, as well as a normal ciliary apparatus and a visible hyperchromatic supranuclear stria (an increase in the number of SNS-positive cells was demonstrated) (Figure 6d, Figure 7c,d and Figure 12d). Supranuclear striae are considered a specific cytological marker of the anatomical and functional integrity of ciliated cells [62]. The presence of a supranuclear stria indicates not only the good condition of the ciliated cells, but is also an important indicator of the therapeutic effectiveness of both local and systemic drug treatment [46,47]. The Golgi apparatus and rough endoplasmic reticulum are located within the SNS [50], and its presence is considered an indicator of high protein content and, consequently, good metabolic activity in cells [63]. The increased metabolic activity of columnar cells is also confirmed by the presence of swollen cell nuclei (Figure 7f). It should be emphasized that the normal condition of cilia also indicates the restoration of proper mucociliary transport and, consequently, a reduced risk of bacterial infection, which we confirmed by the absence of bacteria in the analyzed cytological images. Due to the demonstrated renewal of the ciliated epithelium, there were no columnar cells with features of apoptosis in the examined swabs. Small changes indicating programmed cell death referred only to squamous cells (Figure 6b and Figure 12c). The obtained changes are very favorable and indicate a new therapeutic effect of azelastine in the aspect of rapid restoration of the ciliary epithelium, especially when we take into account that under conditions of frequent chronic inflammatory processes, it is unlikely to obtain a normal ratio between different types of cells in the nasal mucosa, since the normal turnover of the ciliary cell takes three weeks [20,53].

Swabs of patients with NAR/VMR after azelastine treatment showed an almost two-fold increase in neutrophils and a slight increase in lymphocytes and monocytes (Figure 8c, Figure 9f and Figure 13b). Such results can be explained by the hypersensitivity of the nasal mucosa in VMR patients caused by seasonal changes in temperature and humidity; therefore, in spring and autumn, patients may experience increased symptoms, which is reflected in the cytological picture [55,64]. Moreover, it can be suggested that such a condition may have been caused by the presence of pine pollen grains in the atmosphere during the study, which was confirmed by their identification in the analyzed swabs (Figure 8e and Figure 9a). Pine is a minor sensitizer, but it reaches very high concentrations of pollen grains and has the property of mechanically damaging the mucous membrane of the respiratory tract, which can raise the risk of its hyperreactivity [65,66]. The anti-inflammatory effect of azelastine, similarly to patients with AR, was reflected in the morphology of the ciliated epithelium. An important therapeutic effect of the drug was the stimulation of regenerative mechanisms, as evidenced by the presence of basal cells (Figure 8c) and bi- and multinucleated forms (Figure 8b and Figure 9b). Both goblet cells and columnar cells remained in the correct proportion (Figure 13b). Columnar cells with a normal ciliary apparatus formed larger clusters (Figure 8a,d and Figure 9c), hence the percentage of SNS-positive cells increased significantly compared to the pre-treatment condition, but was slightly lower compared to the AR group (Figure 13d). The significant reduction in the number of squamous cells (Figure 13b) was caused by the increased apoptosis of these cells induced by the action of azelastine (Figure 8f, Figure 9d and Figure 13c), which was a significant difference compared to the AR group. Enhancement of programmed cell death is a known mechanism of action for antihistamines, specifically those belonging to the older generation, such as ketotifen and cetirizine [67], but it has not been described so far for azelastine.

The list of preparations used in the treatment of nonallergic rhinitis basically includes most of the molecules used in the treatment of allergic rhinitis; however, according to current data, patients with NAR turn out to be less sensitive to the therapy [30,68]. Reports on the effect of azelastine in nonallergic rhinitis provide information on the effective control of clinical symptoms, such as sneezing, nasal congestion, and rhinorrhea [69]. The literature highlights the role of azelastine as a good alternative to intranasal corticosteroids, which show efficacy in patients with nonallergic rhinitis with eosinophilia (NARES), but have inconsistent results in patients with VMR [57]. Taking into account the pathogenesis of vasomotor rhinitis, it is speculated that the effectiveness of azelastine is due to its non-receptor effect as an anti-inflammatory and neuroinflammatory blocker [55]. The results of our research confirm the anti-inflammatory activity of azelastine and complement the knowledge of new mechanisms of action of azelastine at the cellular level in vasomotor rhinitis. An important aspect of azelastine’s action, similarly to the group of patients with AR, is the stimulation of regenerative changes and rapid renewal of the proper nasal epithelium, despite the presence of numerous neutrophils.

A very characteristic change observed after the use of azelastine in swabs from both AR and NAR/VMR patients was the intensification of vacuolating changes, mainly in the cytoplasm of columnar cells (Figure 10 and Figure 11). Taking into account that darkly stained contents were visible in the lumen of the vacuole, and these were probably epithelial and inflammatory cells in the apoptotic stage and the remains of apoptotic cells (Figure 10a,b and Figure 11c), it can be concluded that the tested compound induced autophagic processes in epithelial cells. Autophagy plays a key role in inflammation, affecting the development, homeostasis, and survival of inflammatory cells, including macrophages, neutrophils, and lymphocytes, and the elimination of pathogens [70,71,72]. It can also be used to eliminate apoptotic cells or remnants of apoptosis involving macrophages [73]. According to the literature, structural cells may also play an important role “in cleaning up” after apoptosis in the role of non-professional phagocytes [74]. Moreover, the significantly expanded area with regular brightening of the cytoplasm (Figure 10e,f and Figure 11a), as well as the numerous vacuoles observed exactly above the nucleus in the columnar cells (Figure 11b,d), suggest the formation of vacuoles from membranes belonging to organelles located in this area, such as the endoplasmic reticulum and the Golgi apparatus, which is typical of the macroautophagy process. Intensified vacuolization of the cytoplasm of columnar cells (Figure 10c,d and Figure 11e,f) and an increased number of vacuolated cells (AR—75.2, NAR—109) (Figure 12c and Figure 13c) indicate intensification of autophagic processes. In turn, the demonstrated higher number of vacuolated cells in the swabs of NAR/VMR patients can be explained by mucosal hyperreactivity expressed by the previously mentioned increase in the number of neutrophils (Figure 13b). In the analyzed cytological images, attention was drawn to the presence of numerous columnar cells with a perinuclear halo effect (Figure 10c and Figure 11a,c). Since in Heffler’s study [20] the reduction of this feature in the columnar cells was correlated with the severity of the symptoms of rhinitis, its presence should be considered a manifestation of normal cell function and at the same time evidence of the effective action of the drug [21].

In the light of current literature data, autophagy, depending on the cell type, may have unpredictable consequences, such as improvement of symptoms or their worsening [73]. In our study, the induction of autophagy in both studied groups, AR and NAR/VMR, represents a positive aspect of azelastine’s action, considering the totality of the results obtained, especially the presence of hyperchromatic supranuclear striae and perinuclear haloes, important markers of the anatomical and functional integrity of the ciliary cells.

## 5. Conclusions

In summary, the results of our study confirmed the efficacy of azelastine used for topical therapy in patients with both allergic and nonallergic/vasomotor rhinitis. Most importantly, we have demonstrated new mechanisms of action for this compound at the cellular level. Our research shows that azelastine stimulates regenerative processes in the ciliated pseudostratified columnar epithelium, as well as induces autophagy and apoptosis, processes necessary to restore homeostasis in the nasal mucosa. The result of the presented research is also a detailed description of cytological changes in allergic and nonallergic rhinitis, which complements the current knowledge regarding prognostic indicators.

## Figures and Tables

**Table 1:** Patient characteristics (groups highlighted according to nasal cytology).

n	Group AR	Group NAR
Number of patients	10	10
Male/Female	7/3	7/3
Age median	8–14 10.9	7–14 11.1
Symptoms of nose		
rhinorrhea	10	10
congestion	10	10
itching	6	-
sneezing	6	10
Other symptoms		
coughing	5	5
mucus in the throat	-	10
Concomitant diseases		
conjuctivitis	5	-
asthma	3	9
atopic dermatitis	2 *	1
Sensitized allergens		
perennial (mites) pollen grains	10	-

AR, allergic rhinitis; NAR, nonallergic rhinitis; * coexisting symptoms in people with asthma.

## Data Availability

The data that support the findings of this study are available from the corresponding author upon reasonable request.
